# Dataset and ANN model prediction of performance of graphene nanolubricant with R600a in domestic refrigerator system

**DOI:** 10.1016/j.dib.2020.106098

**Published:** 2020-07-30

**Authors:** T.O. Babarinde, S.A. Akinlabi, D.M. Madyira, F.M. Ekundayo, P.A. Adedeji

**Affiliations:** a Department of Mechanical Engineering Science, University of Johannesburg, Johannesburg, South Africa; b Department of Mechanical Engineering, Walter Sisulu University, South Africa; c Department of Industrial Engineering, Wayne State University, United States of America

**Keywords:** Graphene nanolubricant, R600a, Power consumption, Cooling capacity, COP, Experimental data, ANN training data, ANN testing data

## Abstract

This work evaluated the steady state performance of R600a in the base lubricant and graphene nanolubricant. The measuring instruments required and their uncertainties were provided, step by step method and procedures for preparation of graphene nanolubricant concentration and substituting it with the base lubricant in domestic refrigerator system are described. The system temperatures data was captured at the inlet and outlet of the system components. Also, the pressures data was recorded at the compressor inlet and outlet. The data was recorded for 3 h at 30 min interval at an ambient temperature of 27 °C. The experimental dataset, Artificial Neural Network (ANN) training and testing dataset are provided. The artificial intelligence approach of ANN model to predict the performance of graphene nanolubricant in domestic refrigerator is explained. Also, the ANN model prediction statistical performance metrics such as Root Mean Square Error (RMSE) and Mean Absolute Deviation (MAD), Mean Absolute Percentage Error (MAPE) and coefficient of determination (R^2^) are also provided. The data is useful to researchers in the field of refrigeration and energy efficiency materials, for replacing nanolubricant with the base lubricant in refrigerator systems. The data can be reuse for simulation and modelling vapour compression energy system.

**Specifications Table**

**Table:** 

**Subject**	Mechanical Engineering
**Specific subject area**	Energy efficiency materials
**Type of data**	Tables, Text file, Figures
**How data were acquired**	Data collection was from the experimental measurement and mathematical calculations. Measuring instruments employed were thermocouples (K-Type), pressure gauges (Bourdon type), and mass flow metre, a digital weighing scale for nanoparticles measurement and refrigerant mass charge. For the Artificial Neural Network (ANN) model, a computer with MATLAB software was used to develop the ANN model performance prediction.
**Data format**	Raw, analyzed
**Parameters for data collection**	The parameters for data collection were refrigerant temperature at evaporator outlet, condenser outlet, discharge pressure and temperature at the compressor outlet and the mass flow rate of the refrigerant
**Description of data collection**	The temperature of the experiment were collected at the inlet and outlet of each component of the refrigerator (Evaporator, compressor, condenser, and expansion valve). Also, the inlet and outlet pressure were collected at the compressor discharge and suction. The experimental data was collected at an ambient temperature of 27° C, the experiment data was captured by the measuring instruments at 30 min interval for 300 min, the experiment was repeated five times to ensure accuracy. In the ANN, 70% experimental data is used for the data training.
**Data source location**	Department of Mechanical Engineering Science, University of Johannesburg, South Africa
**Data accessibility**	With the article
**Related research article**	T. O. Babarinde, S. A. Akinlabi, D. M. Madyira, and F. M. Ekundayo, Enhancing the energy efficiency of vapour compression refrigerator system using R600a with graphene nanolubricant, Energy Reports. vol. 6, pp. 1–10, 2020. https://doi.org/10.1016/j.egyr.2019.11.031

**Value of the Data  **

• The data provides the designing and sizing of nano-refrigerator system energy efficiency, the lack of real experimental raw data makes much difference between the experimental and predicted value differs significantly. The experimental data presented in this experimental provides an expected difference or variation data and ANN predicted values for designing and sizing of nano-refrigerator system.
• The data explains the procedures for investigating and interpreting measured data, calculation and mathematical analysis for performance of nanolubricant in refrigerator system which can be used by researcher in the field of refrigeration and air-conditioning systems and technicians for replacing nanolubricant with the base lubricant in refrigerator systems.
• The ANN model data provides traing and testing data for predicting the performance of graphene nanolubricant in domestic refrigerator system which can be used for other artificial intelligence modelling approach
• The date provides the optimization of graphene nanolubricant concentrations in domestic refrigerator using R600a and this can be applicable to other hydrocarbon refrigerants.
• The data explains the energy performance of an enhanced domestic refrigerator using R600a in graphene nanolubricant.

## 1 Data description

**Fig. 1:**
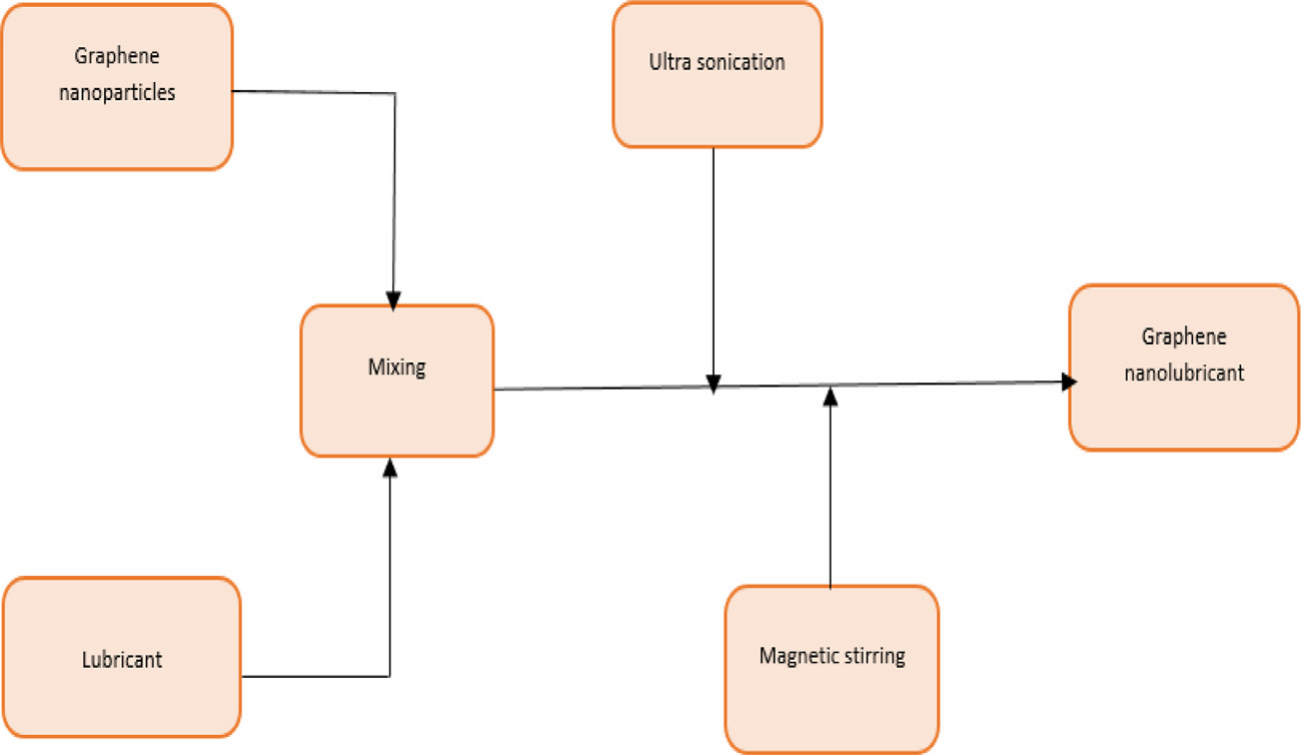
Modified graphene-nanolubricant preparation flow chart (Babarinnde et al. 2020).

**Fig. 2:**
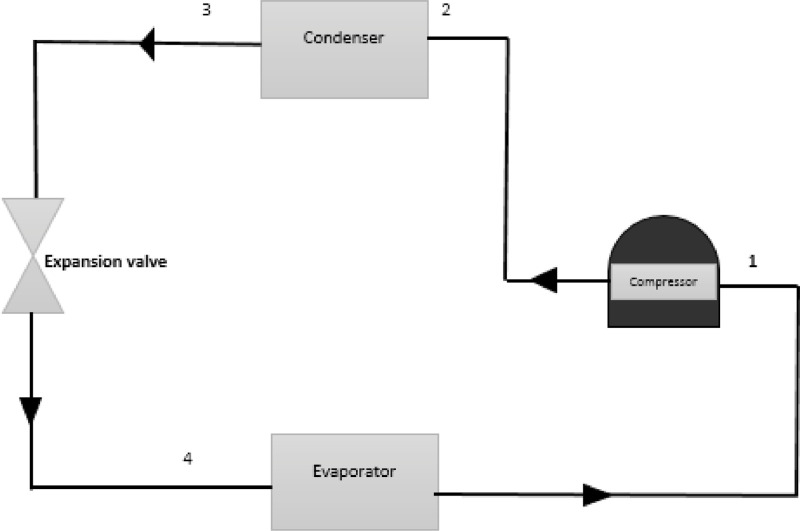
Diagram of the experimental setup.

**Fig. 3:**
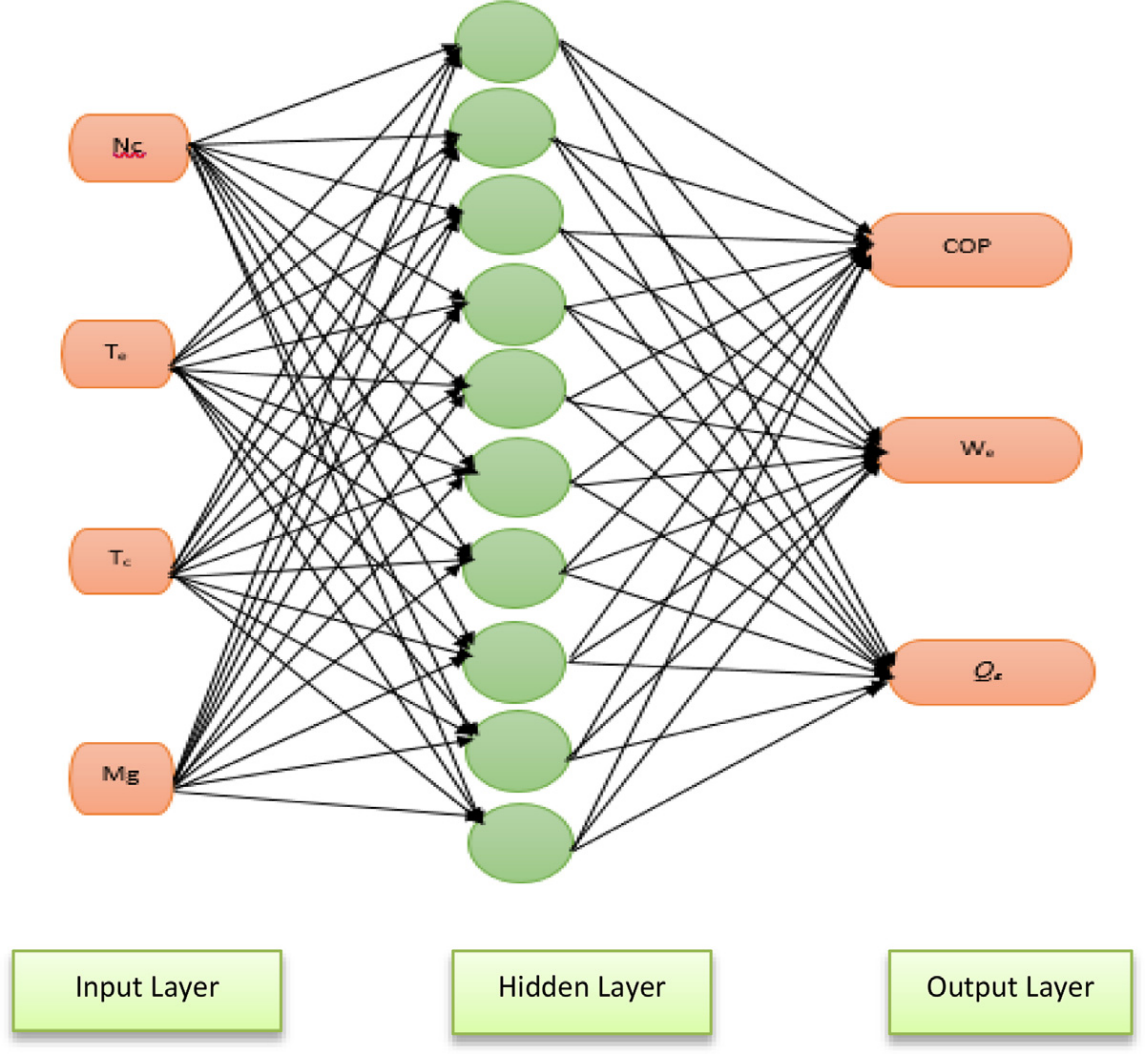
ANN Architecture for the performance prediction of the system.

The experimental set up and the raw data set obtained from graphene nanolubricant with R600a experiment is presented [1]. Fig 1 describe the two step use to prepare the graphene nanolubricant [1]. Fig 2 describes the inlet and outlet of each component of the system and the test point where each data is captured. Fig 3 presents the ANN architecture of the performance prediction of the system with input and output data values. Table 1 provided the data set obtained from obtained from different test point of the experimental set which are R600a mass charge (g), nanolubricant concentration (g/L), temperature ( °C) at outlet of the evaporator and condenser, pressure (MPa) at the suction and discharge line of the compressor and the R600a mass flow rate (kg/s). Table 2 shows the data set of the R600a mass charge and nanolubricant concentration with enthalpy (kJ/kg) of the evaporator, compressor and the condenser and the R600a mass flow rate. Table 3 provides the experimental data set of the performance of the system, the data set includes R600a mass charge, nanolubricant concentration, evaporator cooling capacity (KW), compressor power consumption (kW) and COP of the system. Table 4 describes the experimental data set of the training and testing data set for the system performance. Table 5 shows the statistical analysis such as R-value, RMSE, MAD, MAPE, and Mean STD and number iteration of the predicted dataset.

**Table 1:** Experimental data of temperatures and pressure of the system at steady state.

Mass charge (g)	Nc (g/L)	T_1_ ( °C)	P_2_ (MPa)	T_3_ ( °C)	m_*Ref*_(kg/s)
50	0	−5	0.64	47	0.00075
50	0.2	−10	0.42	31	0.00071
50	0.4	−9	0.54	49	0.00077
50	0.6	−9	0.59	45	0.00077
60	0	−3	0.64	47	0.00071
60	0.2	−13	0.40	30	0.00077
60	0.4	−10	0.42	31	0.00071
60	0.6	−9	0.60	45	0.00075
70	0	0	0.70	46	0.00062
70	0.2	−9	0.53	40	0.00077
70	0.4	−7	0.52	39	0.00074
70	0.6	−7	0.52	39	0.00071

**Table 2:** Experimental data of enthalpy of the system at steady state.

Mass charge (g)	Nc (g/L)	h_1_ (kJ/kg)	h_2_ (kJ/kg)	h_3_(kJ/kg)	m_*Ref*_ (kg/s)
50	0	547.63	657.0967	314.24	0.00075
50	0.2	540.93	634.1694	273.71	0.00071
50	0.4	542.27	641.2310	293.74	0.00077
50	0.6	542.27	644.9973	309.07	0.00077
60	0	550.31	665.9438	314.24	0.00071
60	0.2	536.96	621.5055	271.24	0.00077
60	0.4	540.93	634.0286	273.71	0.00071
60	0.6	543.27	650.070	309.07	0.00075
70	0	554.34	693.0497	311.65	0.00062
70	0.2	542.27	639.9323	296.28	0.00077
70	0.4	544.95	645.0851	293.74	0.00074
70	0.6	544.95	656.2176	293.74	0.00071

**Table 3:** Experimental data of performance of the system at steady state.

Mass charge (g)	Nc (g/L)	Qe (kW)	We (kW)	COP
50	0	0.1742	0.0821	2.1
50	0.2	0.1891	0.0662	2.9
50	0.4	0.1869	0.0762	2.5
50	0.6	0.1809	0.0791	2.3
60	0	0.1677	0.0821	2.1
60	0.2	0.2054	0.0651	3.2
60	0.4	0.1887	0.0661	2.9
60	0.6	0.1762	0.0801	2.2
70	0	0.1415	0.0860	1.7
70	0.2	0.1897	0.0752	2.5
70	0.4	0.1868	0.0741	2.5
70	0.6	0.1779	0.0790	2.3

**Table 4:** Data showing the training dataset and testing dataset of the system at steady state.

Number of data	Experimental Qe (kW)		ANN Predicted Qe (kW)	Experimental Wc (kW)		ANN Predicted Wc (kW)	Experimental COP		ANN Predicted COP
1	0.1869		0.183651	0.0762		0.076303	2.5		2.5000016
2	0.1887	Training	0.190030	0.0661	Training	0.066335	2.9	Training	2.8999998
3	0.1415	data	0.149533	0.0860	data	0.086006	1.7	data	1.7000003
4	0.1762		0.181589	0.0801		0.078838	2.2		2.2168017
5	0.1779		0.185425	0.0790		0.076668	2.3		2.2999981
6	0.1809		0.179934	0.0791		0.078712	2.3		2.2922565
7	0.1887		0.189977	0.0752		0.076019	2.5		2.5000027
8	0.1677		0.176872	0.0821		0.082792	2.1		1.9271949
9	0.1891	Test	0.187151	0.0662	Test	0.070743	2.9	Test	2.7383306
10	0.1868	data	0.169837	0.0741	data	0.079441	2.5	data	1.9918204
11	0.2054		0.190221	0.0651		0.066044	3.2		2.9070025
12	0.1742		0.177551	0.0821		0.083255	2.1		1.8987417

**Table 5:** Performance evaluation of the ANN models.

Parameter	COP		Wc (kW)		Qe (kW)	
	Training	Testing	Training	Testing	Training	Testing
R-Value	0.98647	0.95348	0.98356	0.95856	0.96644	0.63664
RMSE	0.0614	0.3204	0.001	0.0036	0.0056	0.0115
MAD	0.0381	0.1096	7.96E-04	0.0019	0.004	0.0084
MAPE (%)	1.1662	11.1605	0.9307	4.2316	2.761	4.8562
Mean STD	0.0619	0.1549	1.10E-03	0.0023	0.0046	0.0099
No of Iterations	8		6		8	

## 2 Experimental design, materials and methods

The graphene nanolubricant was prepared with a mineral oil and graphene nanoparticles. The mineral oil has a density of 0.914 at 15 °C and viscosity of 32 cSt and 4.4 cSt at 40 °C and 100 °C respectively. The graphene nanoparticles used as additive in the lubricant has a specification of 2nm-8 nm as specified by the manufacturer (Aldrich). Each graphene nanolubricant sample (0.2 g/ L, 0.4 g/L and 0.6 g/L) was prepared with 1 L of mineral oil.

The magnetic stirrer was used to stir the graphene nanolubricant together for 45 min. The Ultrasonic homogeniser was use to homogenized the graphene nanolubricant together for 180minutes under 15–20 °C temperature range [1,2].

The R600a refrigerant used has zero ODP and GWP of 3. The refrigerator used as test rig is a domestic vapour compression system of 70 litres volume capacity with hermetic compressor of 100 W power ratings, expansion valve, air-cooled condenser of 9.8 m length and capillary tube length of 1.5 m.

Each nanolubricant concentration was tested in 50, 60 and 70 g. The R600a mass charges were introduced into the compressor of the system with charging scale. The evacuation and flushing of the system were also carried out for each experiment to ensure better accuracy. The temperature readings were taken at each inlet and outlet of the refrigerator components with thermocouples. The two pressure gauges were connected to compressor to measure the suction and discharge pressure of the compressor.

The experiment was carried out and repeated for five times at an interval of 30 min for 300 min at an ambient temperature of 27 °C. The measurement of the uncertainty of the thermocouple and pressure gauge instrument were ± 3 °C and ± 1% respectively. Fig. 2 represents the schematic-diagram of the experimental set-up. The experimental output readings were used to evaluate the performances of the system using Refprop version 9.0 [3]. Performances such as cooling capacity (Q_e_), power consumption (W_c_), and COP were considered.

The performance such as cooling capacity (*Q_e_*), power consumption (*W_C_*) and COP of R600a in graphene nanolubricant were analysed according to Babarinde et al. [4] using Eq. (1)–(3)

The *Q_e_* was calculated using Eq. (1)

(1) $$Q_{e}=m_{Ref}{\left(h_{1}-h_{4}\right)}_{evap}$$

Where m_*Ref*_*, h_1_*, and *h_4_* represent the mass the mass flow rate, enthalpy of the refrigerant at the evaporator outlet and inlet of the refrigerator respectively. At steady state the enthalpy at the condenser outlet *h_3_* is equal enthalpy at the evaporator inlet *h_4,_* therefore*, h_3_=h_4_.*

The *W_c_* was calculated using Eq. (2)

(2) $$W_{c}=m_{Ref}{\left(h_{2}-h_{1}\right)}_{Comp}$$

Where *h_2_* enthalpy at the compressor outlet

The *COP* is calculated using Eq. (3)

(3) $$COP=\frac{Q_{e}}{W_{c}}$$

A feed forward network was designed and train using Levenberg Marquardt back propagation with 10 neurons at the hidden layer. The normalization of the data was achieved using Eq. (4) where the input and output matrix were processes with minimum and maximum row value between −1 and 1.

(4) $$Y=\frac{\left[y_{max}-y_{min}\right]\times \left[x-x_{min}\right]}{\left[x_{max}-x_{min}\right]}+y_{min}$$

Where Y is the normalized data, *y_max_* is the maximum value of data range, *y_min_* is minimum value of data range, *x* the value to be normalized, *x_min_* is the minimum value of data normalized and *x_max_* is the maximum value of data normalized.

The dataset was divided into two parts, the training and testing data. The training data used was approximately 70% of the whole data. Backward propagated training technique was chosen because of the existence of the data paucity. The weight adjustment was also carried out for each iteration until stopping criteria was achieved. For the network, premium was placed on converge at global optimal region.The stopping criteria includes the maximum time is attained, maximum number of validation increase, minimum performance is attained, maximum number of iteration training is attained and the maximum gradient magnitude is reached.

The experimentally determined model outputs were compared with the ANN predicted values according to Olatunji et al. [5] and statistical performance metrics such as Root Mean Square Error (RMSE) and Mean Absolute Deviation (MAD), Mean Absolute Percentage Error (MAPE) and coefficient of determination (R^2^) . These metrics were calculated as follows:

Coefficient of Determination *(R^2^):*

(5) $$R^{2}=1-\frac{\sum_{k=1}^{N}\left[y_{k}-{\hat{y}}_{k}\right]}{\sum_{k=1}^{N}\left[y_{k}-{\bar{y}}_{k}\right]}$$

Root Mean Square Error *(RMSE):*

(6) $$RMSE=\sqrt{\frac{\sum_{k=1}^{N}{\left[y_{k}-{\hat{y}}_{k}\right]}^{2}}{N}}$$

Mean Absolute Deviation *(MAD):*

(7) $$MAD=\frac{1}{N}\sum_{k=1}^{N}\left|y_{k}-\bar{y}\right|$$

Mean Absolute Percentage Error *(MAPE):*

(8) $$MAPE=\frac{1}{N}\sum_{k=1}^{N}\left|\frac{y_{k}-{\hat{y}}_{k}}{y_{k}}\right|\times 100\%$$

where the *y_k_* is the observed value, ${\hat{y}}_{k}$ is the predicted value, and $\bar{y}$ is the mean of the observed.

## Declaration of Competing Interest

None

## Appendix Supplementary materials

Image, application 1
